# Anthocyanins in Red Jasmine Rice (*Oryza sativa* L.) Extracts and Efficacy on Inhibition of Herpes Simplex Virus, Free Radicals and Cancer Cell

**DOI:** 10.3390/nu14091905

**Published:** 2022-05-01

**Authors:** Boonpa Suantai, Kanyaluck Jantakee, Thida Kaewkod, Sirikwan Sangboonruang, Thararat Chitov, Yingmanee Tragoolpua

**Affiliations:** 1 Department of Biology, Faculty of Science, Chiang Mai University, Chiang Mai 50200, Thailand; boonpa_suantai@cmu.ac.th (B.S.); kanyaluck_j@cmu.ac.th (K.J.); thida_kaewkod@cmu.ac.th (T.K.); thararat.chitov@cmu.ac.th (T.C.); 2 Graduate School, Chiang Mai University, Chiang Mai 50200, Thailand; 3 Division of Clinical Microbiology, Department of Medical Technology, Faculty of Associated Medical Sciences, Chiang Mai University, Chiang Mai 50200, Thailand; sirikwan.sang@cmu.ac.th; 4 Research Center in Bioresource for Agriculture, Industry, and Medicine, Faculty of Science, Chiang Mai University, Chiang Mai 50200, Thailand; 5 Center of Excellence in Materials Science and Technology, Chiang Mai University, Chiang Mai 50200, Thailand

**Keywords:** anthocyanins, anticancer, antioxidant activity, antiviral activity, herpes simplex virus, red jasmine rice

## Abstract

Rice is one of the most important food crops in many countries, with nutritional value and health benefits. In this study, the ethanolic and aqueous extracts of red jasmine rice from Chiang Mai, Thailand were examined for their anthocyanins and phenolic contents. The antioxidant and antiviral activity against herpes simplex virus type 1 (HSV-1) and type 2 (HSV-2), as well as anticancer activity, were investigated. The total anthocyanins content of 708.03 ± 11.56 mg Cy-3-glc equivalent/g extract, determined from the ethanolic extract, was higher than the aqueous extract. However, the aqueous extract showed the highest total phenolic compound of 81.91 ± 0.51 mg GAE/g extract. In addition, the ethanolic extract demonstrated higher antioxidant activity than aqueous extract using DPPH, ABTS, and FRAP assays by 28.91 ± 3.26 mg GAE/g extract, 189.45 ± 11.58 mg 24 TEAC/g extract, and 3292.46 ± 259.64 g FeSO_4_/g extract, respectively. In the antiviral assay, it was found that the ethanolic extract of red jasmine rice could inhibit HSV-1 more effectively than HSV-2 when treated before, during, and after the viral attachment on Vero cells, with 50% effective doses of 227.53 ± 2.41, 189.59 ± 7.76, and 192.62 ± 2.40 µg/mL, respectively. The extract also demonstrated the highest reduction of HSV-1 particles at 4 h after treatment and the inhibition of HSV-1 replication. The ethanolic extract exhibited a higher toxicity level than the aqueous extract, as well as the potential to induce DNA fragmentation by intrinsic and extrinsic apoptosis pathways on the Caco-2 cells. These findings suggest that red jasmine rice extract demonstrates nutritional value and biological activity on HSV, free radicals, and cancer cell inhibition.

## 1. Introduction

Rice (*Oryza sativa* L.) has been a main food in most countries, particularly Asian ones, and it now feeds more than half of the world’s people. Rice is genetically diverse, with two major subgroups, including the *indica* and *japonica* varietal groups [1]. Pigmented rice grains contain varieties of chemical compounds, amino acids, and essential oil compounds that are distributed in their bran layer. An abundance of anthocyanins was observed in the dark purple, black, and red color of rice, which is located in the layers of pericarp [2]. Pigmented rice has been used in traditional Chinese medicine to treat anemia, promote blood circulation, remove blood stasis, improve kidney function, treat diabetes, and improve vision [3]. A variety of beneficial substances can be found in pigmented rice, including anthocyanins, proanthocyanidins, ferulic acid, γ-oryzanols, and other phytochemicals. These bioactive compounds are often prized for their consumer health benefits and properties, including antioxidant, anticarcinogenic, anti-allergic, anti-inflammation and anti-atherosclerosis properties [4]. Recently, phenolic chemicals found in black and red rice bran extracts were linked to the neuroprotective benefits of these colored rice grains in human neuron-like cells [5]. Moreover, black rice had the ability to mitigate antioxidant enzyme and antioxidative stress activities in mice C57BL/6 cells and human HepG2 cells [6]. In addition, anti-inflammatory and antioxidant activities were observed in purple rice extracts [7,8]. Interestingly, pigmented rice consumption provided protection against human diseases and had a good effect on the immune system [9]. It was also reported that women who consumed foods rich in insoluble fiber, such as brown rice, benefited from gallstone protection [10,11]. Moreover, the research found that the diet of purple and brown rice could control the levels of blood glucose by demonstrating anti-hyperglycemic activity in type 2 diabetes patients [12].

Viral pathogenesis is recognized as one of the most important causes of illness and mortality worldwide, including herpes simplex virus (HSV), hepatitis B, hepatitis C virus, human immunodeficiency virus, and SAR-coronavirus type 2 infections [13]. HSV infects millions of people around the world, causing diseases such as herpes genitalis, herpes labialis, encephalitis, and keratitis, all in various age groups with high infection rates. More than 90% of adults have been infected with at least one or more herpes viruses [14]. HSV belongs to the family *Herpesviridae*, which has a linear double-strand DNA genome. The viral genome is enclosed by an icosahedral capsid, which is surrounded by a group of tegument proteins and a lipid bilayer envelope containing membrane proteins and glycoproteins. The virus in the family of *Herpesviridae* is divided into three subfamilies: *Alphaherpesvirinae, Betaherpesvirinae*, and *Gammaherpesvirinae*. HSV-1 and HSV-2 are members of the subfamily *Alphaherpesvirinae*. HSV-1 can cause orolabial disease. HSV-2 is an important sexually transmitted disease and causes genital herpes. Both HSV-1 and HSV-2 remain latent within sensory-neural ganglia and may reactivate, causing recurring infections at primary sites due to stress, hormonal change, and radiation. Currently, acyclovir (ACV) serves as the drug for treatment of these viral infections, deactivating the DNA polymerase in the viral DNA replication step [15]. Because of the high cost of this synthetic antiviral agent, serious side effects, and the inevitable development by HSVs of drug-resistant strains after long-term treatment, the exploration of natural remedies with anti-viral properties should be considered. In Thailand, medicinal herbs and plants are often used for cooking ingredients. They are also used for the treatment of various infections based on traditional folk wisdom. Additionally, Thai plants are sources of many beneficial compounds that may be useful against HSVs [16,17]. A previous study found that natural products contain potential anti-HSV agents, which provide several advantages, such as reductions in side effects, resistant viruses, and toxicity [18]. Moreover, the anti-cancer compounds of Thai plant extracts were studied in cervical, lung, ovarian, and breast cancers [19,20,21]. The ubiquitous availability and knowledge of local plants deems them an easy and reliable source that would be trusted for use in patients.

The incidence of cancer is a global health problem, with the highest mortality rates resulting from colon cancer. Colon cancer is the second most frequent cancer after breast cancer, and it is the most common cancer in males and the second-most common cancer in females. Conventional treatments, such as chemotherapy, radiation, and surgery, are recommended [22,23]. Currently, synthetic anticancer drugs may generate strong side effects due to toxicity on tissues or normal cells. Therefore, the discovery of new plant extracts or substances needs to occur for the prevention and treatment of cancers. Phytochemical and bioactive compounds from Thai plant extracts have a significant impact on the expression of pharmacological and biological activities [24]. Thai plant extracts were shown to inhibit many cancers, including liver, colon, and cervical cancers [25,26]. Flavonoids are included in the phenolic compound groups that can provide potential health benefits and aid in the prevention of several chronic diseases, including cancer. Moreover, γ-oryzanols, γ-tocotrienol, and anthocyanins, from pigmented black rice and purple rice, exert anti-carcinogenesis properties and inhibit the growth of cancer cells, such as prostate, colon, and liver cancers [27,28,29]. In addition, anthocyanins from black rice could inhibit breast cancer cells in vitro and in vivo [30].

Therefore, the new anti-HSV and anti-cancer properties derived from Thai plant extracts were interesting to study. Accordingly, this research aims to demonstrate the nutritional value and the biological properties of the red jasmine rice (*O. sativa* L.) crude extract on the inhibitory activities of HSV infection, free radicals, and cancer cell.

## 2. Materials and Methods

### 2.1. Reagents and Chemicals

2,20-azinobis-(3-ethylbenzothiazolin-6 sulfonic acid, ABTS) was purchased from Fluka, Germany. Gallic acid monohydrate, 2,2-diphenyl-1-picrylhydrazil (DPPH), carboxymethyl cellulose, and 6-hydroxy-2,5,7,8-tetramethyl-chroman-2-carboxylic acid (Trolox) were obtained from Sigma-Aldrich (St. Louis, MO, USA). 2,4,6-tri(2-pyridyl)-s-triazine, TPTZ, Folin–Ciocalteu reagent, and quercetin dehydrate were purchased from Merck (Billerica, MA, USA). Ferrous sulfate, sodium acetate, and potassium chloride were purchased from Qrec, New Zealand. Streptomycin and penicillin were obtained from Gibco (Grand Island, NY, USA). Crystal violet was purchased from Loba, India. Acyclovir was obtained from Siam Pharmaceuticals, Thailand. 3-(4,5-Dimethylthiazol-2-yl)-2,5-Diphenyltetrazolium Bromide, (MTT) was obtained from Bio Basic, Canada. TRIzol Reagent was obtained from Ambio (Carlsbad, CA, USA).

### 2.2. Preparation of Red Jasmine Rice Extract

Red jasmine rice was obtained from an organic farm in Chiang Mai, Thailand. Samples were soaked in 95% ethanol (RCI Labscan, Bangkok, Thailand) for three days at room temperature before being extracted in distilled water for three hours at 45 °C, with the ratio of extracts and solvent as 100 g/L. The extracts were filtered before evaporation using a rotary evaporator (Heidolph, Schwabach, Germany) and were then dried with a lyophilizer (Labconco, Kansas City, MO, USA). The percentage of yield was calculated according to Equation (1) and dry powder of the extracts was dissolved in dimethyl sulfoxide (DMSO) and stored at −20 °C until use. Moreover, gamma aminobutyric acid (GABA) and γ-oryzanol were determined using high performance liquid chromatography (HPLC), Central Laboratory, Faculty of Agriculture, Chiang Mai University.

(1) $$\mathrm{Percentage}\;\mathrm{of}\;\mathrm{yield}\;(\%)=\;\;\frac{\mathrm{Weight}\;\mathrm{of}\;\mathrm{red}\;\mathrm{jasmine}\;\mathrm{rice}\;\mathrm{after}\;\mathrm{extraction}\;\left(g\right)\;}{\mathrm{Weight}\;\mathrm{of}\;\mathrm{red}\;\mathrm{jasmine}\;\mathrm{rice}\;\mathrm{before}\;\mathrm{extraction}\;\left(g\right)}\times 100$$

### 2.3. Antioxidant Activities

#### 2.3.1. DPPH Radical-Scavenging Activity

The anti-free radical of rice extracts was determined using the DPPH method [31]. The solution of 100 mM DPPH radical was prepared in methanol. The extracts were prepared with methanol at various concentrations. The extracts and standards at 0.5 mL were mixed with DPPH solution (1.5 mL) in methanol at various concentrations. After incubation at ambient temperature in the dark for 20 min, the mixture was measured at the wavelength of 517 nm. Gallic acid was selected as a standard compound. The capability to scavenge the DPPH radical was calculated according to Equation (2). The anti-DPPH radical activity was expressed in milligrams of gallic acid per gram of extract.

(2) $$\mathrm{DPPH}\;\mathrm{radical}\;\mathrm{scavenging}\;\mathrm{activity}\;(\%)=\;\frac{A\;\mathrm{control}-A\;\mathrm{sample}\;}{A\;\mathrm{control}}\;\text{×}\;100$$

#### 2.3.2. ABTS Radical Cation Scavenging Activity

The antioxidant capacity of the extracts was analyzed using a spectrophotometer by ABTS method [31]. ABTS radical cation (blue-green color) was first prepared by oxidizing 7 mM ABTS (5 mL) with 140 mM potassium persulfate (88 µL) in water, which was stored in the dark at ambient temperature for 12–16 h before use. The ABTS+ solution was adjusted with methanol to an absorbance of about 0.700 ± 0.050 at 734 nm. The extracts of 5 µL were mixed 500 µL of with ABTS reagent at various concentrations. After incubation in the dark at ambient temperature for 10 min, the mixture was detected at the wavelength of 734 nm. Trolox was selected as a standard. The results were expressed in terms of milligrams of trolox equivalents (TEAC) per gram of extract. The percentage of scavenging ABTS radical capacity was computed using the following Equation (3):

(3) $$\mathrm{ABTS}\;\mathrm{radical}\;\mathrm{cation}\;\mathrm{decolorization}\;\mathrm{activity}\;(\%)=\;\frac{A\;\mathrm{control}-A\;\mathrm{samples}}{A\;\mathrm{control}}\;\text{×}100$$

#### 2.3.3. Ferric Reducing Antioxidant Power (FRAP) Assay

Ferric reducing antioxidant power was detected using a FRAP reagent, which performed the reductants in a redox-linked colorimetric method [32]. The composition of the FRAP reagent was prepared from 300 mM acetate buffer pH 3.6, 20 mM ferric chloride solution, 10 mM 2,4,6-tri(2-pyridyl)-s-triazine, TPTZ in 40 mM HCl, and distilled water and stored in a water bath at 37 °C. The reaction was prepared from the mixture of 0.5 mL of the extract and 1.5 mL of the FRAP reagent. After 15 min of incubation in the dark at ambient temperature, the absorbance was measured with a spectrophotometer at 593 nm. In the FRAP assay, the ferrous sulphate (FeSO_4_) was used as a positive control. The calibration curve of the FeSO_4_ was generated within a range of 10 to 100 µg/mL (R^2^ = 0.9998). The ferric reducing antioxidant activity was calculated and the data were expressed as gram FeSO_4_ per gram extract (g FeSO_4_/g extract).

### 2.4. Determination of Total Anthocyanins Content

The total anthocyanins content in red jasmine rice extract was investigated by the differential pH method [33]. The extracts were mixed in potassium chloride buffer (0.025 mM KCl) with pH 1.0 and 0.4 mM sodium acetate buffer (CH_3_COONa) with pH 4.5 (1:100 *v*/*v*) at various concentrations. After 20 min of incubation at room temperature, the combination was measured at 510 nm and 700 nm. The total anthocyanin content per gram of extract was expressed as milligrams of cyaniding 3-glucoside equivalent. The following Equation (4) was employed to compute the total anthocyanins content

(4) $$\mathrm{Total}\;\mathrm{anthocyanin}\;\mathrm{content}\;(\mathrm{mg}\;\mathrm{Cy}\text{-}3\text{-}\mathrm{glc}/g\;\mathrm{Extract})=\frac{A\left(\mathrm{total}\right)\times \;\mathrm{MW}\;\times \;\mathrm{DF}\;\times 1000}{\;\varepsilon \times 1}$$

➢ A_total_ = (A_510nm_ − A_700nm_) _pH 1.0_ − (A_510nm_ − A_700nm_) _pH 4.5_
➢ MW = Molecular weight of cyanidin-3-glucoside (Mw = 484.84)
➢ DF = Dilution factor
➢ ε = Molar extinction coefficient = L × mol^–^^1^ × cm^–^^1^ (anthocyanin = 26,900)
➢ L = Length of cell path (1 cm)

### 2.5. Determination of Total Phenolic Content

Folin–Ciocalteu is a combination reagent containing phosphomolybdate and phosphotungstate that is generally used to investigate the concentration of phenolic and polyphenolic compounds [32]. The Folin-Ciocalteu colorimetric method was employed to investigate the total phenolic components in red jasmine rice extract. The extract and gallic acid were mixed with Folin-Ciocalteu reagent at various concentrations. After incubation in the dark at ambient temperature for 5 min, 5% sodium carbonate was used to neutralize the reaction before incubation in the dark at ambient temperature for 1 h. At a wavelength of 725 nm, the content of total phenolic compounds was determined and represented as milligrams of gallic acid equivalent per gram of extract.

### 2.6. Cell Line and Viruses

Vero cells and Caco-2 cells were cultured in Dulbecco’s modified Eagle’s medium (DMEM) from Gibco (Grand Island, NY, USA), supplemented with 10% heat inactivated fetal bovine serum from Thermo Fisher Scientific (Waltham, MA, USA), streptomycin (100 µg/mL) and penicillin (100 units/mL), and maintained in a humidified atmosphere of 37 °C at 5% CO_2_.

Standard herpes simplex virus strains F and G (HSV-1 and HSV-2) were cultured on Vero cells, and virus titers were measured using a plaque titration method. The virus stock was collected and kept at −80 °C for future study. Viral titer was measured by virus titration assay before use.

### 2.7. Evaluation of Viral Titer by Plaque Titration Assay and Plaque Reduction Assay

#### 2.7.1. Plaque Titration Assay

Vero cells were cultured using DMEM supplemented with 10% FBS and added into 24-well plates at 1.5 × 10^5^ cells/well. The cells were incubated for 24 h to form monolayers before HSV infection. HSV was serially diluted ten-fold in DMEM before infection to the cell monolayer. After viral infection for 1 h, the cells were rinsed with FBS and overlay medium containing 1.5% sodium carboxymethyl cellulose, and in growth medium was added to each well. After 2–3 days of incubation, the plaque formation was stained by adding 0.1% crystal violet in 1% ethanol for 20 min. The percentage of inhibition was expressed as the average number of plaques from triplicate wells. The viral titers were expressed as PFU/mL [17].

#### 2.7.2. Plaque Reduction Assay

The Vero cell monolayer was cultured in a 24-well plate and infected with 80–100 PFU of HSV for 1 h on a rocking platform at room temperature. The suspension of virus was discarded after incubation and rinsed with phosphate buffered saline (PBS). Then, non-toxic concentrations of extract were added to the infected cells. At 2–3 days post-infection, the plaque formation was detected after staining with 0.1% crystal violet in 1% ethanol for 20 min. The percentage of inhibition was expressed as the average number of plaques from triplicate wells. The EC_50_ value is an effective concentration achieving 50% of viral plaque inhibition after treatment with the tested compound [17].

### 2.8. Antiviral Activities

The antiviral activity of red jasmine rice extract was investigated by plaque reduction assay. The extracts were added at various phases of the viral infection step to evaluate the antiviral mode of action, as set out below.

#### 2.8.1. Effect of Red Jasmine Rice Extracts on HSV upon Treatment before Viral Attachment

The monolayer of Vero cells was cultivated in a 24-well plate and incubated for 24 h. The extracts were then placed in a well with a Vero cell monolayer for 1 h at ambient temperature. The mixture was then discarded and rinsed with PBS before adding HSV. Finally, the overlay media, which contained 1.5% carboxymethyl cellulose in growth medium, with the ratio of 1:3, was dispended and incubated for 2–3 days at 37 °C in a 5% CO_2_ incubator. Plaques were stained with 0.1% crystal violet in 1% ethanol for 20 min and counted to compare with the infected cell control without extract. The plaque reduction was used to calculate the percentage of HSV inhibition, as compared to the viral control [17].

#### 2.8.2. Effect of Red Jasmine Rice Extract on HSV upon Treatment during Viral Attachment

Non-toxic concentrations of red jasmine rice extract and 100 µL of HSV at the titer of 1 × 10^4^ PFU per mL were added into Vero cells cultured in 24-well plates at the same time. The mixture was then incubated at ambient temperature for 1 h. After removing the mixture, it was rinsed with PBS. The overlay media, which included 1.5% carboxymethyl cellulose in growth medium with the ratio of 1:3 was added and cultured for 2–3 days at 37 °C in 5% CO_2_ incubator. The plaques were stained with 0.1% crystal violet in 1% ethanol for 20 min and counted to compare with cell control [17].

#### 2.8.3. Effect of Red Jasmine Rice Extract on HSV upon Treatment after Viral Attachment

Vero cells were cultivated for 24 h in a 24-well plate until they formed a monolayer. The cells were then infected with 100 µL of HSV at the titer of 1 × 10^4^ PFU/mL and incubated at ambient temperature for 1 h. After viral adsorption, the HSV supernatant was removed and the infected cells were rinsed with PBS to remove the free viral particles. The red jasmine rice extracts were then added to the infected cells. The overlay media, which included 1.5% carboxymethyl cellulose in growth medium, with the ratio of 1:3, was then added and cultured for 2–3 days at 37 °C in 5% CO_2_ incubator. The plaques were stained with 0.1% crystal violet in 1% ethanol for 20 min. The plaques were counted and compared to the ACV and viral control group, which were not treated by the extracts. A dose response curve was also used to determine the ED_50_ value, which represented 50% suppression of plaque formation [17].

#### 2.8.4. Effect of Red Jasmine Rice Extract on HSV Replication

The monolayer of Vero cells was grown in a six-well plate. Then the cells were infected with HSV at 1 × 10^6^ PFU/ mL and incubated at ambient temperature for 1 h. After viral absorption, the infected cells were rinsed twice with PBS to remove unattached virus. The highest non-toxic concentration of the red jasmine rice extract was added to the infected cells. After treatment, the infected cells were incubated at 37 °C in a 5% CO_2_ incubator and collected at 0, 6, 12, 24, 30, and 36 h after virus replication. Finally, the infected cells were frozen and thawed twice, and the virus was stored at −80 °C. Viral titer was observed by plaque titration assay and compared with ACV, antiviral drugs, and the virus control [17].

#### 2.8.5. Effect of Red Jasmine Rice on Virus Particles

HSV stock was treated with the highest non-toxic concentration of red jasmine rice extract in the ratio of 1:1. The treatment was incubated for 0, 1, 2, 3, and 4 h at ambient temperature to allow the virus particles in the extract to be inactivated, while treated with DMEM as viral control. After that, the mixture was stored at −80 °C to investigate the residual virus by plaque titration assay. The plaques were counted to compare with the virus control [17].

### 2.9. Anticancer Activities

#### 2.9.1. Cytotoxicity of Caco-2 Cells by MTT Assay

The in vitro cytotoxicity of red jasmine rice extract was evaluated on a monolayer of Caco-2 cells by MTT cell viability assay [34]. Vero cells (1.5 × 10^5^ cells/well) and Caco-2 cells (1 × 10^4^ cells/well) were seeded into 96-well plates using the DMEM medium, supplemented with 10% FBS and incubated at 37 °C in a 5% CO_2_ incubator for 24 h. After that, the medium was discarded and the extracts were serially diluted two-fold from 39.06 to 5000 µg/mL. The red jasmine rice was added to each well at different concentrations with a final volume of 100 µL. using untreated cells and DMSO as control. After incubation at 37 °C in 5% CO_2_ for 72 h, 2 mg/mL of MTT reagent was dispended to each well and incubated at 37 °C for 4 h. Finally, the formazan crystal that was formed by the living cells was dissolved by DMSO. The absorbance was detected at 540 nm with a reference wavelength of 630 nm using a microplate reader. The CD_50_ value was computed using the mean ± SD of three independent studies.

#### 2.9.2. DNA Fragmentation Analysis

The TUNEL assay (Terminal Deoxynucleotidyl Transferase and Fluorescein-labeled dUTP) is a standard method for detecting DNA fragmentation caused by apoptosis. Caco-2 cells were treated with red jasmine rice extract and were observed for DNA fragmentation using TUNEL assay (Roche, Mannheim, Germany). In brief, the untreated and the Caco-2 cells treated with extract for 48 h were collected before fixation with the fixative solution (0.1% Triton-X 100 and 4% Paraformaldehyde in purified water) for 10 min at ambient temperature. The cells were rinsed with ice cold PBS. After that, the reaction solution containing enzyme solution and DNA labeling solution were added to the cells and incubated for 1 h at 37 °C in dark. Then, nuclei dye solution was added into the cells and incubated for 5 min at ambient temperature in the dark. The nuclei dye solution was discarded and rinsed with PBS. Finally, PBS solution was added to the cells before observation using a fluorescence microscope [35].

#### 2.9.3. Apoptotic Gene Expression in Caco-2 Cells Using Quantitative Real Time Polymerase Chain Reaction (qRT-PCR)

Caco-2 cells were incubated with red jasmine rice extract. Then, total RNA was isolated with TRIzol Reagent. Pure RNA that showed an A260/A280 ratio of 1.5–2.1 was used for the next step. The total RNA was reverse transcribed into cDNA using ReverTra Ace^®^ qRT PCR Master Mix (TOYOBO, Osaka, Japan) and the cDNA were maintained at −20 °C until use. PCR amplifications were detected using SensiFAST SYBR kit (BIOLINE, London, UK) with specific primers for *caspase-3, caspase-8*, and *caspase-9* genes (Table 1). Moreover, the glyceraldehyde-3-phosphate dehydrogenase (GAPDH) gene was used as an endogenous control to normalize the differences in the amount of total RNA in each sample. The PCR reactions were amplified with an initial denaturation at 95 °C for 2 min, followed by 40 cycles at 95 °C for 5 s and 60 °C for 30 s. The results were obtained using qRT PCR and a related fold change in mRNA expression was analyzed. All of the experiments were carried out in three independent trials.

### 2.10. Statistical Analysis

All data were shown as the mean ± SD of the three independent experiments. The statistical analysis of the data was executed using one-way analysis of variance (ANOVA) on SPSS 22.0 for Windows and using GraphPad Prism vision 9.3 (GraphPad Software, San Diego, CA, USA). Statistical significance was set as a value of *p* < 0.05.

## 3. Results

### 3.1. Anthocyanins, Phenolic Compounds and Antioxidant Activity of Red Jasmine Rice Extracts

The yields of aqueous and ethanolic extracts of red jasmine rice after extraction were 5.76% and 4.03%, respectively. The ethanolic extract contained high anthocyanins level of 708.03 ± 11.56 mg Cy-3-glc equivalent/g extract and a total phenolic content of 81.91 ± 0.51 mg gallic acid/g extract, which was higher than the aqueous extract (Table 2). The antioxidant activity of the extracts was investigated by three different methods. The ethanolic extract was found to have the highest antioxidant activity in DPPH, ABTS, and FRAP assays, with 28.91 ± 3.26 mg gallic acid equivalents per gram extract (mg GAE/g extract), 189.45 ± 11.58 mg Trolox equivalents antioxidant capacity (TEAC) per gram extract (mg TEAC/g extract), and 3292.46 ± 259.64 g FeSO_4_/g extract (g FeSO4/g extract), respectively (Table 3). Moreover, the compounds in the ethanolic extracts were analyzed and found to contain γ-oryzanol at 1989.19 mg/g of extract and gamma aminobutyric acid, GABA of 1.45 mg/g of extract.

### 3.2. Anti-HSV Activity

**Figure 1:**
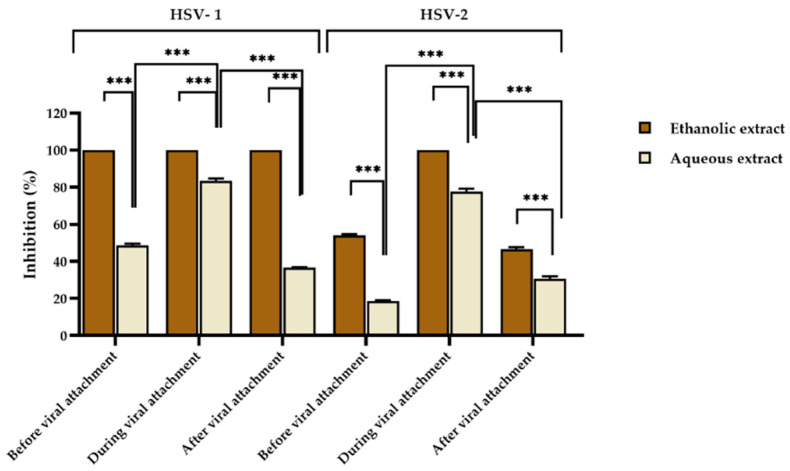
Inhibitory effect of aqueous red jasmine rice extract at 1250 µg/mL and ethanolic extract at 625 µg/mL on HSV-1 and HSV-2 before, during, and after viral attachment. (*** indicated, *p* < 0.0001).

Non-toxic concentrations of red jasmine rice extracts were analyzed for antiviral activity. In the study of antiviral activity, the concentrations that did not affect the growth of the host cells were chosen at 625 and 1250 µg/mL for ethanolic and aqueous extracts, respectively. By using a plaque reduction assay, the extracts were tested for inhibition of HSV infection before, during, and after viral attachment to Vero cells. It was found that the ethanolic extract had the ability to inhibit HSV-1 at all stages of viral infection by 100%, whereas the aqueous extract could interfere HSV-1 infection during viral attachment by 83.44 ± 1.32%. However, the rice extract could inhibit HSV-1 less than 50% when treated before and after viral attachment by 48.66 ± 0.94% and 36.61 ± 0.34%, respectively (Figure 1).

Moreover, the ethanolic extract of red jasmine rice showed complete inhibition on HSV-2 when treated during viral attachment, whereas when treated with the ethanolic extract of red jasmine rice before and after viral attachment, the inhibitions of HSV-2 were 54.03 ± 0.75% and 46.67 ± 0.95%. In addition, when HSV-2 was treated with the aqueous extract during viral attachment, the virus was inhibited by 77.73 ± 1.56%. The virus was also inhibited before and after viral attachment by 18.62 ± 0.43% and 30.60 ± 1.37%, which were less than 50% (Figure 1).

This research revealed that the ethanolic extract of red jasmine rice exhibited the greatest inhibition on HSV-1. HSV-2 was inhibited more than 50% by both extracts during viral attachment. Moreover, the effective doses of the extracts at 50% (ED_50_) were computed by dose-response curves and expressed as 50% inhibition of plaque formation. The ethanolic extract of red jasmine rice was shown to be more efficient than the aqueous extract, since the ED_50_ values of the ethanolic extracts were less than the aqueous extract, as shown in Table 4. Acyclovir (ACV) at ED_50_ values of 1.72 and 2.83 µg/mL was selected as a positive control to inhibit HSV-1 and HSV-2, respectively.

**Figure 2:**
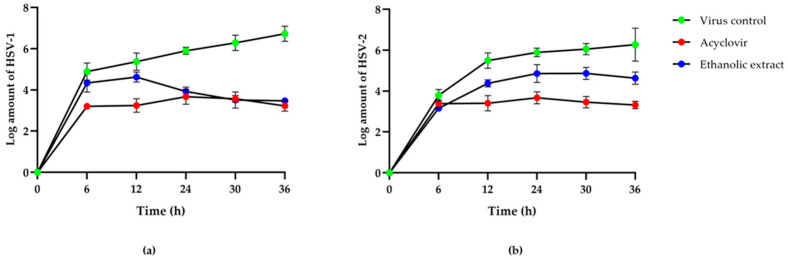
Log titers of HSV-1 (**a**) and HSV-2 (**b**) at 0, 6, 12, 24, 30, and 36 h after treatment with ethanolic extract of red jasmine rice at 625 µg/mL, compared with antiviral agent, acyclovir and virus control.

A high anti-HSV activity was found from the ethanolic extract of red jasmine rice. Therefore, this study examined the inhibitory effect of extracts on HSV viral replication at 0, 6, 12, 24, 30, and 36 h after HSV infection, which was compared with the HSV inhibition by ACV and the virus control. The results demonstrated that HSV titers reduced in time when treated with these red jasmine rice extracts. After 12 h of viral infection, the HSV-1 titer was reduced more than the HSV-2 titer after treatment with the ethanolic extract of red jasmine rice. The inhibitory effect of HSV-1 replication was also observed after reducing the log virus titer by 3.25 ± 0.14 PFU/mL (Figure 2a). The reduction of log HSV-2 titer was shown after treatment with ethanolic extract by 1.64 ± 0.15 PFU/mL, when compared with viral control at 36 h after treatment (Figure 2b). Thus, the results indicated that the ethanolic extract of red jasmine rice revealed more anti-HSV-1 activity than HSV-2 replication.

**Figure 3:**
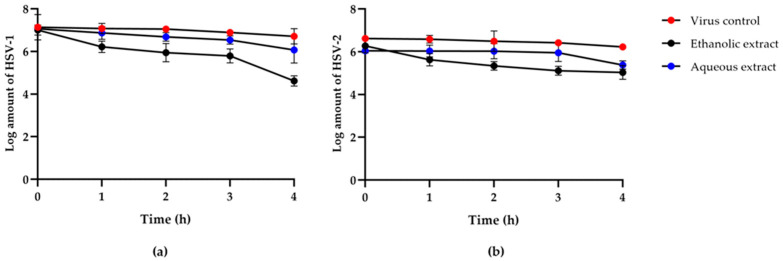
Log titers of HSV-1 (**a**) and HSV-2 (**b**) titer at 0, 1, 2, 3, and 4 h after treatment with the extracts from ethanolic and aqueous extracts of red jasmine rice, compared to viral control.

The plaque titration assay was used to measure the virus from direct inactivation of HSV-1 and HSV-2 by red jasmine rice extracts at 1, 2, 3, and 4 h after viral inactivation, and the data were compared to a viral control group. The results showed that HSV particles were inactive after being treated with extracts in a time and dose-dependent manner in all experiments. Both the ethanolic and aqueous extracts of red jasmine rice were effective on the viral particles. The results revealed that the highest reduction number of plaque was observed after treatment at 4 h. The ethanolic extract of red jasmine rice showed the greatest inactivation on the HSV-1 particle. The log reduction of HSV-1 titer was 2.40 ± 0.30 PFU/mL, compared to the viral control. However, the log reduction of HSV-1 titer was 0.99 ± 0.07 PFU/mL after treatment with aqueous extract of red jasmine rice, when compared to the viral control group (Figure 3a). In addition, the ethanolic extract of red jasmine rice reduced HSV-2 replication, and the log reduction of HSV-2 titer was 1.24 ± 0.10 PFU/mL. HSV-2 was inhibited by the aqueous extract of red jasmine rice, with a log reduction of 0.67 ± 0.14 PFU/mL (Figure 3b).

### 3.3. Anti-Cancer Activity of Red Jasmine Rice Extracts

#### 3.3.1. Cytotoxicity of Caco-2 Cells of Red Jasmine Rice Extracts

**Figure 4:**
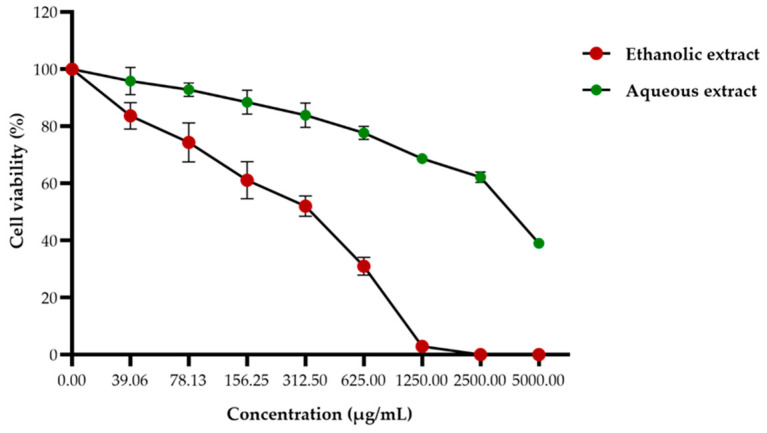
Effect of the ethanolic and aqueous extracts of red jasmine rice on Caco-2 cell viability. The cells were treated with various concentrations of 39.06 to 5000 µg/mL for 48 h and toxicities of the cells after treatment were investigated by MTT assay.

The effect of red jasmine rice extracts on Caco-2 cell viability were investigated after treatment with several concentrations of ethanolic or aqueous extracts by MTT assay. The cell viability of Caco-2 cells was decreased in a dose-dependent manner. At 48 h after treatment, the 50% inhibitory concentrations (IC_50_) of ethanolic and aqueous extracts were 372.56 ± 15.54 and 3177.60 ± 92.36 µg/mL, respectively (Figure 4). This finding demonstrated that the ethanolic extract of red jasmine rice revealed a greater toxicity to inhibit the growth of Caco-2 cells than red jasmine rice aqueous extract.

**Figure 5:**
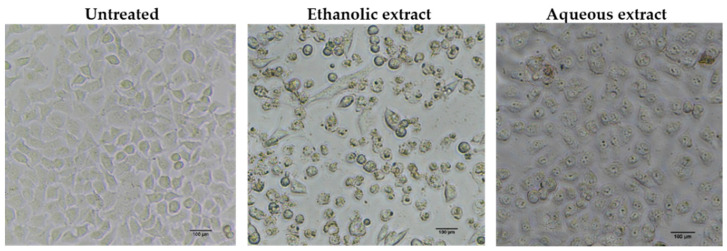
Morphological characteristics of apoptosis were detected in the cells after treatment with 250 µg/mL of red jasmine rice ethanolic extract and 3000 µg/mL of red jasmine rice aqueous extract for 48 h, when compared to untreated cells.

**Figure 6:**
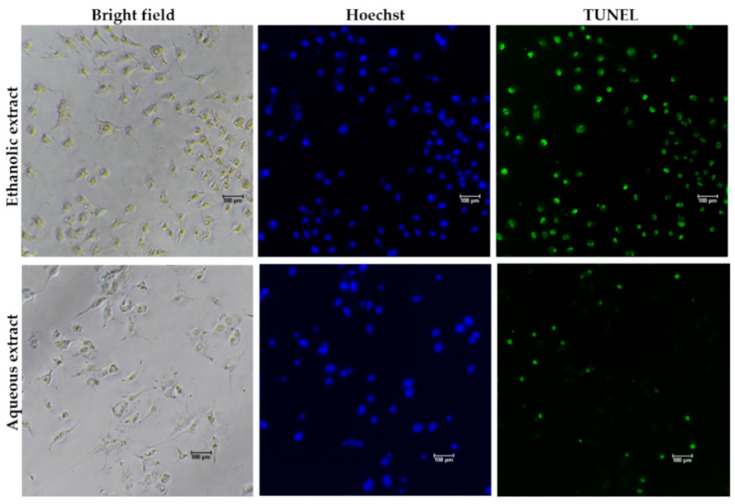
DNA fragmentation in treated Caco-2 cells after 48 h of treatment with 250 µg/mL of red jasmine rice ethanolic extract and 3000 µg/mL of red jasmine rice aqueous extract after investigation by TUNEL assay and fluorescent microscope.

To determine whether the growth inhibitory activity of red jasmine rice extract was induced through the mechanism of apoptosis, the morphological characteristics of apoptosis were further investigated. The results demonstrated that the treated cells exhibited loss of cell attachment, cell shrinkage, and rounding when compared to the untreated cell (Figure 5). Moreover, a TUNEL assay was carried out to confirm the DNA fragmentation, which is one of the characteristics indicating apoptotic cell death. The results showed that TUNEL-positive cells were observed to be fluorescent green after treatment with red jasmine rice extract for 48 h (Figure 6). These cells indicated that the ethanolic and aqueous extracts of red jasmine rice triggered DNA damage during the late stage of apoptosis in Caco-2 cells.

#### 3.3.2. Effect of Red Jasmine Rice Extract on Apoptotic-related Gene Expression

**Figure 7:**
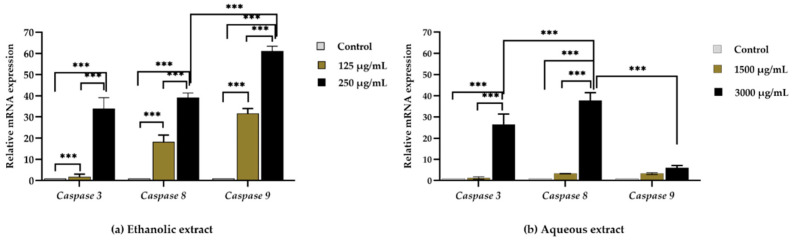
The qRT-PCR analysis of apoptosis-related gene after 3 h of treatment of Caco-2 cell with red jasmine rice ethanolic extract (**a**) and aqueous extract (**b**), compared with untreated cell control. Data are expressed as mean ± SD (*n* = 3 independent assays). (*** indicated, *p* < 0.0001).

Caco-2 cells were selected to determine the anticancer effect of extracts from red jasmine rice on the apoptotic mRNA expression. The qRT-PCR was used to evaluate the relative mRNA expression levels of *caspase-3*, *caspase-8*, and *caspase-9*. The mRNA expression of apoptotic genes increased in a dose-dependent manner after treatment with ethanolic extract of red jasmine rice (Figure 7a). However, mRNA expressions of *caspase-3* and *caspase-8* were upregulated after treatment by the aqueous extract of red jasmine rice (Figure 7b). Therefore, the red jasmine rice extracts had the ability to induce through different apoptosis pathways. The results indicated that the ethanolic extract of red jasmine rice could induce the extrinsic and intrinsic apoptosis pathways via the induction of *caspase-3, caspase-8*, and *caspase-9* genes. In contrast, the aqueous extract of red jasmine rice revealed the upregulation of *caspase-3* and *caspase-8* genes, which are related to the induction of extrinsic apoptosis pathway.

## 4. Discussion

Several non-pigmented and pigmented rice had bioactive compounds that have been developed in order to improve their application in food and pharmaceutical supplements. The main bioactive compounds are found in the aleurone layer of the rice grain and referred to as the bran/germ part. The pigmented rice bran consists of various compounds, such as anthocyanins, proanthocyanidin, and polyphenolic compounds. Red rice has traditionally been regarded as a nutritious diet in Asian countries such as Japan, China, and Korea; it has gained attention due to the health benefits of phytochemicals [4]. Several studies have shown the health advantages of red rice by way of its potential antioxidant, anti-inflammation, and anticancer properties. Natural sources could also present active components to protect the impact of free radicals on cells. Similarly, this study found that red jasmine rice extract showed the highest level of antioxidants. The antioxidant potential of red rice and its crude extract were investigated, and the results indicated that the use of colored rice has the potential to boost antioxidant capacity, both in vitro and in vivo [38]. Moreover, it was revealed that high levels of proanthocyanidin, anthocyanins, γ-oryzanol, and phenolic compounds possess potential biological activities. The results showed that red jasmine rice displayed a high amount of γ-oryzanol content. γ-oryzanol is the major component from rice and rice bran extracts; it has been confirmed as safe for human and animal consumption, with minimal side effects, and is used as a dietary supplement in the United States [39].

γ-oryzanol has been shown to inhibit cancer growth [40], reduce cholesterol levels [41], and protect against the development of gastric ulcers [42]. Moreover, γ-oryzanol can protect against inflammation and oxidative stress [43]. A previous study reported that red jasmine rice from Thailand contains a high value of γ-oryzanol in crude extract and demonstrates anti-invasive activity on a fibrosarcoma cell line [44]. Additionally, anthocyanins are one of the most common groups of natural pigments found in plants, and they are abundant in red jasmine rice extract that exhibits orange, blue, red, and purple colors. Anthocyanins and proanthocyanidin were discovered to be the principal functional components in many rice types, especially pigmented rice, which are strongly responsive against reactive oxygen species (ROS) [45]. Not only does naturally pigmented rice harbor highly beneficial antioxidant activities, but the colored rice also shows strong anti-inflammatory properties. Anthocyanins had the ability to control the secretion and expression of inflammatory factors by inactivation of the transcription factor NF-κB, through multiple pathways, to provide their anti-inflammation function. For example, cyanidin-3-glucoside (C-3-G), petunidin-3-glucoside, and delphinidin-3-glucoside inhibited the stimulation of NF-κB. It can also prevent the activation of STAT3 and inhibit the action of COX-2 and inducible NO synthase (iNOS), as well as the synthesis of their products NO and PGE2 [46,47,48,49,50]. It has been reported that the proanthocyanidin of red rice inhibits the secretion of iNOS, NO, TNF-α, IL-6, and COX-2 in LPS-treated Raw 264.7 cells by the inhibition of NF-κB activation, AP-1, and the MAPKs signaling pathway [51]. Anthocyanins from purple rice were found to have an inhibitory effect on heart inflammation and hypertrophy. In rats, purple rice also reduced heart fibrosis and supported cardiac function [52]. Additionally, anthocyanins derived from wild blueberries showed anti-inflammation in Caco-2 cells by reducing NF-κB activation in the presence of the pro-inflammatory stimulus IL-1ß [53]. Moreover, in other plants, anthocyanins from *Hibiscus* sp. and lowbush blueberry (*Vaccinium myrtillus*) have been applied in treatments of diarrhea, liver dysfunction hypertension, and microbial infections [54,55]. Tsuda et al. (2003) demonstrated that anthocyanins from purple corn effectively reduced adipose tissue and body weight in mice that were provided a high-fat diet. The report suggested that obesity and diabetes can be prevented by anthocyanins [56]. Anthocyanins from black currants could enhance rhodopsin regeneration, which is one of the mechanisms that enhance visual acuity [57].

Interestingly, this research demonstrated that the ethanolic extract of red jasmine rice revealed the highest percentage of HSV-1 and HSV-2 inhibition during virus attachment to the cells. The ethanolic extracts of red jasmine rice may be a promising candidate for the treatment of HSV infection. The active components of the extract might interact with the viral outer surface proteins. Thus, the compound may interfere with the viral entry and penetration into the cell. A recent report showed that delphinidin-3-rutinoside, belonging to the anthocyanin family of *Solanum melongena,* inhibits HSV-1 replication, reduces viral proteins expression, and reduces NADPH oxidase 4, NOX4 expression [58]. Similarly, the total anthocyanin extract from the strawberry and *Lamiaceae* family inhibits the virus adsorption and replication stage of HSV infection [59,60]. The compound from the fruit *Terminalia chebula* exhibited activity against HSV-2 by inhibiting virus attachment and penetrating the host cells [61]. HSV-1 and HSV-2 were inhibited by oligomeric proanthocyanidins from *cranberry (Vaccinium macrocarpon)* [62]. In addition, the extract prepared from Japanese rice-koji miso displayed inhibitory activity on hepatitis A virus (HAV) replication that could enhance the GRP78 (glucose-regulated protein 78) expression, which inhibited HAV replication [63]. El-Shiekh et al. (2020) reported that anthocyanins extracted from *Hibiscus schizopetalus* flower could inactivate HSV-1, adenoviruses, and Coxsackie B4 virus [64]. Some anthocyanin derived from small red bean extracts (*Vigna angularis*) could interfere the infectivity and the early stage of rabies virus infection [65]. Recently, Ana et al. (2021) reported that red algae (*Solieria filiformis*) extract, which has high anthocyanins, showed activity against HSV-1 infection [66]. Moreover, elderberry fruit containing anthocyanins could inhibit SARS-CoV-2 viral budding from the host cell [67].

The significance of rice grain diet is clarified by active phytochemicals in the inhibition of many types of cancer cells by presenting a cytotoxic effect, which is supported by previous studies. This study found that the extracts of red jasmine rice could induce the extrinsic and intrinsic apoptosis pathways of Caco-2 cells through the stimulation of *caspase-3, caspase-8*, and *caspase-9* genes. Anthocyanins-rich extracts were shown to inhibit Caco-2 cell growth. The stimulation of caspase 3, caspase 8, and caspase 9 activity interfered with the basic cellular function, including cell cycle progression and apoptosis. Similarly, HepG2 hepatocellular carcinoma cells’ apoptosis was induced by a proanthocyanidin-rich extraction derived from bran and red rice germ [68]. Another report presented the activity of purple rice extract against HepG2 cells and LNCaP cells (human prostate adenocarcinoma cells). The highest inhibition was shown on HepG2 cells, which triggered apoptosis through the mitochondrial pathway, with the loss of mitochondrial transmembrane potential and the stimulation of caspase-3 and caspacse-9 [8].

Similarly, Yoon et al. (2014) reported that HepG2 cells were inhibited by black rice extract. The stimulation of pro-survival signal proteins (Akt and ERKs) protected HepG2 cells against oxidative damage. Furthermore, red jasmine rice could interfere with the invasion of MDA-MB-231 human breast cancer and HT1080 fibrosarcoma cells. Proanthocyanidin, γ-oryzanol, and γ-tocotrienol fractions from red jasmine rice extract could decrease matrix metalloproteinase-2 and metalloproteinase-9 secretions in cells [44]. In addition, after treating the Jurkat cell line with fermented brown rice, the extract could inhibit cell proliferation and promote cancer cell death via the apoptotic pathway [69]. In other plants, anthocyanins from strawberry and grape extracts performed as anticancer agents by stimulating apoptosis in colon cancer cells [70]. Additionally, anthocyanins from purple potato could inhibit colon cancer proliferation by increasing apoptosis through the mitochondria pathway in vivo, thereby decreasing the number of colon cancer stem cells [71]. Similarly, Hsu et al., (2012) revealed that anthocyanins from purple-shoot tea extract could reduce the proliferation of human colorectal carcinoma cells (HT-29 and COLO 320DM cells) by inhibiting the cycle of cell progression during the G_0_/G_1_ phase and stimulating apoptotic death. In 2012, Huang et al. found that anthocyanin from mulberry could activate the apoptosis of gastric cancer cells via the external receptor p38/Fas/FasL/Caspase-8 pathway [72]. Zhang et al. (2019) demonstrated that anthocyanins from black raspberry extract were active against colon cancer by inhibiting the expression of β-catenin [73,74]. Thus, in this study, high contents of anthocyanin and γ-oryzanol in red jasmine rice may be involved as bioactive compounds for the inhibition of cancer cell proliferation, which may be a prospective novel dietary agent to prevent and treat colon cancer. Moreover, this study also supports research of other properties of γ-oryzanol and anthocyanins [75,76]. Overall, this study suggests that red jasmine rice may be developed as a new antiviral and anticancer agent in the future.

## 5. Conclusions

Rice contains nutritional value and health benefits. The ethanolic extract of red jasmine rice demonstrated high total phenolic content, anthocyanins, and the greatest antioxidant activity using DPPH, ABTS, and FRAP assays. The highest anti-HSV-1 activity was found in the ethanolic extract of red jasmine rice. The HSV-1 viral particles and viral replication were also inhibited more than HSV-2. Moreover, this extract caused toxicity on Caco-2 cells via the intrinsic and extrinsic apoptosis pathway. This study is the first report of anti-HSV activity from the extract of red jasmine rice. Therefore, the new knowledge from this study demonstrates the nutritional value and biological activity of red jasmine rice from Thailand on HSV, free radicals, and cancer cell inhibition.

## Figures and Tables

**Table 1:** Oligonucleotides used in qRT PCR amplifications from extracts treated Caco-2cell.

Target Genes	Sequence of PCR Primers (5′–3′)	References
*caspase-3*	Forward: TGTTTGTGTGCTTCTGAGCC	[36]
	Reverse: TCAAGCTTGTCGGCATACTG	
*caspase-8*	Forward: GTGGAGGAAAGCAATCTGTC	[36]
	Reverse: TATTAGCCCTGCCTGGTGTCT	
*caspase-9*	Forward: GACTCCCTCGAGTCTCCAGAT	[36]
	Reverse: GACTCCCTCGAGTCTCCAGAT	
GAPDH	Forward: GAAGGTGAAGGTCGGAGTC	[37]
	Reverse: GAAGATGGTGATGGGATTTC	

**Table 2:** Total phenolic and anthocyanins contents of red jasmine rice extracts.

Extracts	Total Phenolic Content (mg GAE/g Extract)	Total Anthocyanins Content (mg Cy-3-glc/g Extract)
Ethanolic extract	81.91 ± 0.51 ^a^	708.03 ± 11.56 ^b^
Aqueous extract	65.29 ± 0.67 ^a^	356.24 ± 15.42 ^a^

Each value in the table is represented as mean ± SD (*n* = 3); ^a,b^ indicate significant difference (*p* < 0.05).

**Table 3:** Antioxidant activities of red jasmine rice extracts from DPPH, ABTS, and FRAP assays.

Extracts	Antioxidant Activities		
	DPPH (mg GAE/g Extract)	ABTS (mg TEAC/g Extract)	FRAP (g FeSO_4_/g Extract)
Ethanolic extract	28.91 ± 3.26 ^b^	189.45 ± 11.58 ^b^	3,292.46 ± 259.64 ^b^
Aqueous extract	7.96 ± 0.28 ^a^	84.69 ± 4.33 ^a^	934.18 ± 68.13 ^a^

Each value in the table is represented as mean ± SD (*n* = 3); ^a,b^ indicate significant difference (*p* < 0.05).

**Table 4:** The 50% effective dose of ethanolic and aqueous extracts of red jasmine rice using plaque reduction assay.

Viruses	Stage of Viral Infections	ED_50_ (µg/mL)	
		Ethanolic Extract	Aqueous Extract
HSV-1	Before viral attachment	227.53 ± 2.41 ^a^	-
	During viral attachment	189.59 ± 7.76 ^a^	874.75 ± 15.22 ^b^
	After viral attachment	192.62 ± 2.40 ^a^	-
HSV-2	Before viral attachment	298.83 ± 2.12 ^b^	-
	During viral attachment	229.03 ± 1.62 ^a^	831.55 ± 38.50 ^b^
	After viral attachment	-	-

Each value in the table is represented as mean ± SD (*n* = 3); ^a,b^ indicate significant difference (*p* < 0.05).

## Data Availability

The data presented in this study are available on request from the corresponding author.
